# Ocean acidification in a geoengineering context

**DOI:** 10.1098/rsta.2012.0167

**Published:** 2012-09-13

**Authors:** Phillip Williamson, Carol Turley

**Affiliations:** 1 School of Environmental Sciences, University of East Anglia, Norwich NR4 7TJ, UK; 2 Natural Environment Research Council, Swindon SN2 1EU, UK; 3 Plymouth Marine Laboratory, Prospect Place, The Hoe, Plymouth PL1 3DH, UK

**Keywords:** geoengineering, ocean acidification, carbonate chemistry system, pH impacts, carbon dioxide removal, solar radiation management

## Abstract

Fundamental changes to marine chemistry are occurring because of increasing carbon dioxide (CO_2_) in the atmosphere. Ocean acidity (H^+^ concentration) and bicarbonate ion concentrations are increasing, whereas carbonate ion concentrations are decreasing. There has already been an average pH decrease of 0.1 in the upper ocean, and continued unconstrained carbon emissions would further reduce average upper ocean pH by approximately 0.3 by 2100. Laboratory experiments, observations and projections indicate that such ocean acidification may have ecological and biogeochemical impacts that last for many thousands of years. The future magnitude of such effects will be very closely linked to atmospheric CO_2_; they will, therefore, depend on the success of emission reduction, and could also be constrained by geoengineering based on most carbon dioxide removal (CDR) techniques. However, some ocean-based CDR approaches would (if deployed on a climatically significant scale) re-locate acidification from the upper ocean to the seafloor or elsewhere in the ocean interior. If solar radiation management were to be the main policy response to counteract global warming, ocean acidification would continue to be driven by increases in atmospheric CO_2_, although with additional temperature-related effects on CO_2_ and CaCO_3_ solubility and terrestrial carbon sequestration.

## 1. Carbon dynamics in today’s ocean

### (a) The ocean carbon cycle

The ocean exchanges CO_2_ with the atmosphere and provides an important net sink for carbon. Carbon uptake by the ocean has slowed the increase in atmospheric CO_2_ and its associated consequences for the Earth’s climate: without such uptake, atmospheric CO_2_ would now already be approximately 450 ppm [1]. The net ocean uptake (approx. 2 Gt C yr^−1^) is, however, small in terms of the natural fluxes between the reservoirs, representing only about 2 per cent of the total CO_2_ cycled annually across the air–sea interface. Thus relatively minor changes in ocean biogeochemistry or ocean physics affecting carbon fluxes— in either direction—could have a major impact on the magnitude, or even sign, of the net CO_2_ flux and hence on the future climate.

The large natural annual fluxes of CO_2_ between the ocean and the atmosphere are due to a combination of physical and biological processes, the former driven by ocean circulation and the latter involving marine productivity, calcification and particle sinking. Around half of primary production on Earth is carried out by marine phytoplankton—microalgae and photosynthetic bacteria—that require sunlight, nutrients (primarily supplied from deep waters) and dissolved inorganic carbon (DIC; see §1*b*). As phytoplankton consume DIC in the upper ocean, they can cause an undersaturation of dissolved CO_2_, hence driving CO_2_ uptake from the atmosphere. Although most of the carbon fixed through this process is respired within days to months through processing by the marine food web, a small proportion is repackaged into faecal pellets or aggregates that fall through the deep ocean. The carbon in these particles is removed from the atmosphere for decades to centuries, and, for an even smaller proportion which is not remineralized, incorporated in deep-sea sediments for millions of years.

Physical, chemical and biological geoengineering techniques have all been proposed to increase carbon sequestration in the ocean; these are discussed in greater detail in §5.

### (b) The ocean carbonate system

**Figure 1.**
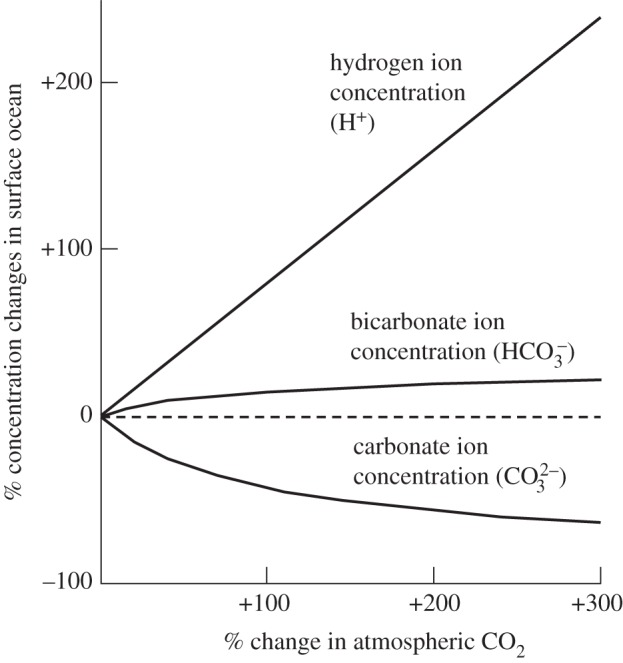
Percentage changes in average global surface ocean ion concentrations resulting from up to a fourfold change (300% increase) in atmospheric carbon dioxide, compared with pre-industrial values and at an assumed uniform and constant upper ocean temperature of 18^°^C. Values for atmospheric CO_2_ change from 280 to 1120 ppm; bicarbonate ions from 1770 to 2120 μmol kg^−1^; carbonate ions from 225 to 81 μmol kg^−1^; and pH from 8.18 to 7.65 (where pH is defined as the negative decimal logarithm of the hydrogen ion activity, and a linear relationship is assumed between activity and concentration). Adapted from Royal Society [3].

DIC is present in seawater in four forms: dissolved carbon dioxide (CO_2_), carbonic acid (H_2_CO_3_), bicarbonate ions (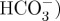 and carbonate ions (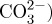. These occur in dynamic equilibrium, reacting with water and hydrogen ions (H^+^). At a mean surface seawater pH of 8.1 and salinity of 35, approximately 91 per cent of the DIC is bicarbonate, with about 8 per cent as carbonate and less than 1 per cent each as dissolved CO_2_ and carbonic acid [2]. Increased CO_2_ in the atmosphere leads to increases in dissolved CO_2_, carbonic acid, bicarbonate and hydrogen ion concentrations, hence pH falls. However, the concentration of carbonate ions decreases, as a result of a reaction between CO_2_ and carbonate. The relative changes in bicarbonate, carbonate and hydrogen ion concentrations in the surface ocean arising from doubling, tripling and quadrupling of atmospheric CO_2_ (compared with pre-industrial values) are shown in figure 1.

The decrease in carbonate ions increases the rate of dissolution of CaCO_3_ minerals in the ocean. The saturation state (*Ω*) is the degree of CaCO_3_ saturation in seawater:

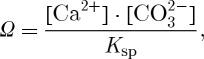

where [Ca^2+^] and [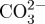] are the *in situ* calcium and carbonate ion concentrations, respectively, and *K*_sp_ is the solubility product for CaCO_3_ (concentrations when at equilibrium, neither dissolving nor forming). Values of *K*_sp_ depend on the crystalline form of CaCO_3_; they also vary with temperature and pressure, with CaCO_3_ being unusual in that it is more soluble in cold water than warm water.

Environments with high saturation states are potentially more suitable for calcifying organisms (plants and animals that produce shells, plates and skeletons of CaCO_3_), since high *Ω* values reduce the energy required for bio-calcification, involving active intracellular regulation of Ca^2+^, H^+^ and inorganic carbon [4], and also help maintain the integrity of mineral CaCO_3_ after its formation [5]. The inclusion of a proteinaceous organic matrix increases shell stability under low pH and low saturation conditions; however, it also increases the metabolic cost of shell formation [6], by up to 50 per cent. Currently, *Ω* is more than 1 for the vast majority of the surface ocean, i.e. seawater is supersaturated with respect to CaCO_3_. However, most of the deep ocean is unsaturated, *Ω*<1, owing to changes in temperature, pressure and the accumulation of biologically produced CO_2_; it is therefore corrosive to unprotected CaCO_3_ structures, and calcareous (micro-) fossils are absent from sediments below the level at which the rate of CaCO_3_ dissolution exceeds the rate of its supply.

The three main mineral forms of CaCO_3_, in order of least to most soluble, are calcite, aragonite and magnesium-calcite; their differences in *K*_sp_ result in each form having different saturation state profiles and saturation horizons, with the aragonite saturation horizon (ASH) being shallower than the calcite saturation horizon (CSH). *Ω* varies with latitude (mostly because of temperature effects), with lowest surface *Ω*_aragonite_ in the Arctic and Southern Oceans currently mostly below 1.5 [7], although with large spatial and seasonal variability. The ASH depth in the North Pacific is less than or equal to 600 m but in the North Atlantic can be more than 2000 m, this difference being due to global circulation patterns affecting CO_2_ values at depth. Increasing atmospheric CO_2_ will cause *Ω* to decrease, and the ASH and CSH levels to move towards the sea surface, as has already occurred in the past 200 years [8]. Most of the Arctic is projected to be undersaturated with respect to aragonite and calcite by approximately 2030 and 2080, respectively, with equivalent values for the Southern Ocean being approximately 2060 and 2100 [7].

There is currently considerable spatial and seasonal variation in ocean surface carbonate system parameters and pH, with the latter varying from 7.6 to 8.2 [3,9,10]. The highest surface pH occurs in regions of high biological production, where dissolved CO_2_ is less than atmospheric levels as a result of DIC being fixed by phytoplankton and exported into deeper water. The lowest open ocean pH values occur in upwelling regions (e.g. west coasts of North America and South Africa, the equatorial Pacific and the Arabian Sea) where mid- and deep waters with high dissolved CO_2_ and low pH are brought to the surface [11]. Seasonally low pH can also occur in coastal waters and estuaries, subject to eutrophication effects, high organic loads and low-pH river inputs.

## 2. Observed chemical and biological changes owing to ocean acidification

### (a) Evidence for anthropogenic ocean acidification

Model-based calculations indicate that, since the industrial revolution (approx. 1800), the release of anthropogenic CO_2_ to the atmosphere and subsequent flux into the ocean has reduced the global average surface pH by approximately 0.1 unit, equivalent to approximately 30 per cent increase in H^+^ concentrations [8]. Since 1990, surface ocean pH has directly been measured or calculated at several locations, with the average recent decrease estimated as 0.0019 pH units per year at the Hawaii Ocean Time-series (HOT; close to the site of long-term atmospheric CO_2_ measurements at Mauna Loa) [12]; 0.0017 per year based on transects in the North Pacific [13]; 0.0012 per year at the Bermuda Atlantic Time-Series (BATS) [14] and 0.0017 per year at the European Station for Time-Series in the Ocean at the Canary Islands (ESTOC) [15]. There can, however, be relatively large interannual variability, likely to be caused by variability in CO_2_ flux rates [16]. Aragonite saturation, calcite saturation and carbonate ion concentrations were measured or estimated in several of these studies; such parameters also showed a marked decline over the last decade.

### (b) Impacts of recent ocean acidification on organisms and ecosystems

At most open ocean locations, the estimated decreases in pH and carbonate ion concentration since the industrial revolution have now exceeded current seasonal variability, with potential impacts (negative or positive) on marine organisms. Field evidence for such effects is, however, inconclusive, owing to a lack of long time-series carbonate chemical data with which biological observations can be correlated [17]. There are also inherent limitations in the interpretation of historical data involving simultaneous changes in many environmental parameters, such as temperature, nutrients, pollutants, food-web structure and local/regional circulation changes.

For example, reductions in the abundance of two species of pteropods (planktonic marine molluscs) and of bivalve larvae are apparent in large-scale survey data for the northeastern Atlantic over the period 1960–2007 [18]. Yet for echinoderm larvae, no consistent changes occurred, and for foraminifera and coccolithophores (data for latter limited to 1990–2007, and not well sampled by the Continuous Plankton Recorder) there is evidence for recent increases in abundance—that may be climate-driven or due to other changes in plankton distributions and biodiversity [19]. The absence of coccolithophores from the Baltic Sea might be because of existing acidification and low saturation conditions (winter *Ω*_calcite_ values less than 1), or because of low salinity [20].

Recent shell thinning has been reported for the planktonic foraminifera *Globigerina bulloides* in the Southern Ocean [21], and other ecological effects of ocean acidification might be expected to initially occur elsewhere where *Ω* values are already low. For example, in upwelling areas along the western coast of North America, where shelf-sea waters can be undersaturated from February to September (with pH values as low as 7.6) [11], and the Pacific coast of Central America. Coral reefs occur in the latter regions, but produce little or no interskeletal pore cement to hold them together and suffer some of the highest erosion rates measured [22]. This is in contrast to the coral reefs in the tropical Atlantic off the Bahamas that live in waters with less CO_2_ and higher pH, and which have a high percentage of interskeletal pore cement (60% occurrence, compared with less than 2% for Galapagos samples) [22].

Field data for more direct effects of reduced pH on warm-water corals are sparse. Nevertheless, reefs in the Red Sea have shown correlated responses in net calcification rate to natural fluctuations in *Ω* and temperature [23], and decreases in net calcification of 14–21% and in growth of 13–30% have been reported over the past approximately 20 years for corals in the Great Barrier Reef [24]. Sea surface temperature is uncorrelated to this decline.

Cold-water corals do not need sunlight and mostly live at depths of 200–2000 m, with their lower depth range closely matching the ASH [25]. However, the ASH has been shoaling at a rate of approximately 1 m yr^−1^ off California [11] and up to 4 m yr^−1^ in the Iceland Sea [26]. The latter causes 800 km^2^ of the deep-sea floor around Iceland, previously bathed in saturated waters, to be newly exposed to undersaturation each year. It is thus likely that cold-water corals are increasingly becoming exposed to corrosive waters, and such deep-water ecosystems might therefore be the most vulnerable to current and future levels of ocean acidification [27]. Although cold-water corals are difficult to study, controlled laboratory experiments indicate that calcification by *Lophelia pertusa*, a long-lived structure-forming species, may be very sensitive to ocean acidification [28].

## 3. A ‘business as usual’ future ocean

### (a) Decadal to century-scale future acidification

If anthropogenic CO_2_ release continues to track the highest emission scenarios used to date for climate projections by the Intergovernmental Panel on Climate Change [1,29], atmospheric CO_2_ will exceed 1000 ppm by 2100. Hydrogen ion concentrations in surface waters would then double (figure 1), resulting in a pH fall of approximately 0.4 since pre-industrial times [3]. If all known fossil fuel reserves were to be used, on a somewhat longer time scale, surface ocean pH would decline by approximately 0.7 compared with pre-industrial levels [30].

Such pH shifts would greatly change CaCO_3_ saturation values. Undersaturation would occur earliest in polar and sub-polar regions [7,8,31,32], and saturation levels would also slowly decline at all depths throughout the global ocean [33,34]. Thus the saturation horizons for both aragonite and calcite would shoal by 100–1000 m, with greatest ecological impact expected for shelf seas in the Pacific, in upwelling regions, and in polar and sub-polar waters [8].

The global mean surface ocean pH predicted for 2050 is likely to be lower than mean surface values previously experienced by marine ecosystems over the last 24 million years, with the current rate of pH change being more rapid than experienced for approximately 60 million years [35–37].

### (b) Potential future impacts on marine organisms and ecosystems

Several hundred experimental studies have been carried out in the past decade, to simulate the impacts of a high CO_2_ world on a wide range of taxonomic groups and biological processes. For reviews, see [8,10,14,17,38–41].

**Figure 2.**
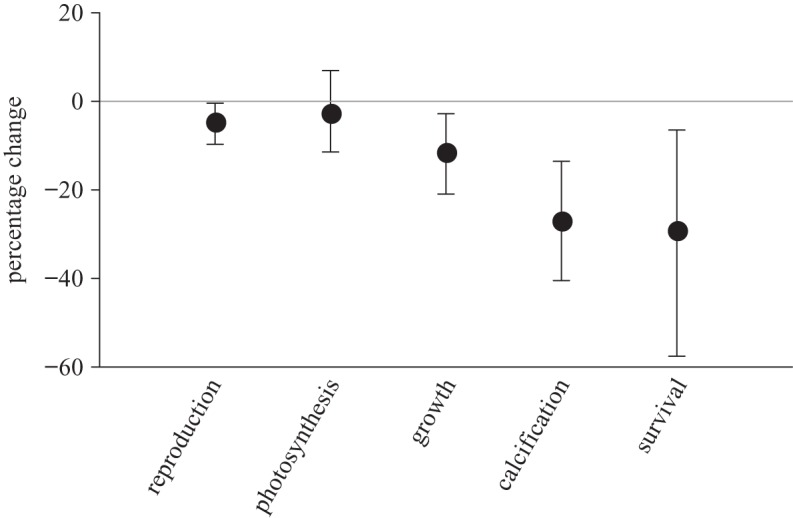
Meta-analysis of the effect of pH decrease by 0.4 units on reproduction, photosynthesis, growth, calcification and survival of a wide range of marine organisms. Mean effect and 95% confidence limits calculated from log-transformed response ratios, here re-converted to a linear scale. Adapted from Kroeker *et al.* [43].

Recent meta-analyses have combined experimental data from different studies, for all organisms [42,43] and for microbes and microbially driven processes [44]. Based on 372 studies, the meta-analysis by Hendriks *et al.* [42] found that calcification was the process most sensitive to ocean acidification. However, because there were positive as well as negative effects for some species and processes, these authors questioned whether marine functional diversity would be much impacted at pH scenario values for 2100. That conclusion has been criticized [43,45] as failing to take account of heterogeneities within groupings, and minimizing the importance of vulnerable life-cycle stages. The meta-analysis by Kroeker *et al.* [43] used more robust methods; they found significant negative effects of a 0.4 pH change on survival, calcification, growth and reproduction, as summarized in figure 2.

Although decreased calcification might be considered an unsurprising impact of ocean acidification [46], some organisms increase calcification under experimental treatments [47], usually at the expense of other physiological processes [48]. There is also high variability in other observed responses, as indicated in figure 2. Such sensitivity differences may reflect species-specific responses to different carbonate chemistry parameters [49] as well as differences in the ability of species and groups to regulate internal pH [38]. Enzyme function, protein phosphorylation and the carrying capacity of haemoglobin for O_2_ are all pH-sensitive, and there is a metabolic cost in regulating pH to maintain these processes.

For all organisms, prolonged exposure to pH values lower (or higher) than evolved optimal conditions will therefore require more energy for internal pH regulation, reducing the energy available for growth, maintenance or reproduction. For example, calcification in corals is costly, requiring 13–30% of energy expenditure compared with tissue growth which requires approximately 8 per cent [50]. Organisms with an active high-metabolic lifestyle such as brachyuran crustaceans, teleost fish and cephalopods may be better adapted to cope with future ocean acidification than those with low-metabolic lifestyles, such as bivalves and echinoderms, although even those with high-metabolic lifestyles may be vulnerable in early life stages [51].

For more sedentary species, effects are likely to be greater and even small changes in physiology or behaviour can produce major changes in population success under competitive environmental conditions. Indirect ecological implications may, however, not be apparent in relatively short term laboratory experiments where food and nutrients are usually abundant, and competitors and predators absent. In such experiments, organisms can eat more to supply the increased energy demand, without trading-off energy for other physiological processes. Overall, the increase in metabolism frequently observed in ocean acidification experiments should be considered a negative, rather than positive impact (although the opposite interpretation has also been made [42]).

Nevertheless, there are marine organisms, mostly photosynthetic, that genuinely do seem to benefit from ocean acidification under experimental conditions. These include seagrasses, some non-calcifying phytoplankton (micro-algae and cyanobacteria) and several other microbial groups (table 1). These might benefit directly, by CO_2_-enhancement of photosynthesis, or indirectly, if predators and competitors are reduced in abundance.

**Table 1.** Summary of probable main effects of future ocean acidification on different groups of marine organisms, mostly based on experimental studies.

group	main acidification impacts
warm-water corals	a relatively well-studied group. The great majority of experiments show that increasing seawater CO_2_ decreases adult coral calcification and growth, and suppresses larval metabolism and metamorphosis [14,52,53]. Although most warm-water coral reefs will remain in saturated waters by 2100, saturation levels are predicted to decline rapidly and substantially; thus, coral calcification is unlikely to keep up with natural bioerosion [22,31,32,54,55]. Interactions with other climatic and anthropogenic pressures give cause for concern [56,57]
cold-water corals	the long-lived nature of cold-water corals, and their proximity to aragonite saturation horizons, makes them vulnerable to future shoaling of the ASH. Approximately 70% of known cold-water coral locations are estimated to be in undersaturated waters by the end of this century [25,27]. Experiments found the effect of pH change on calcification was stronger for fast growing, young polyps [28]
molluscs	significant effects on growth, immune response and larval survival of some bivalves [58–60], although with high inter-specific variability [61–63]. Pteropods seem particularly sensitive [8,64,65] and are a key component of high latitude food webs. Molluscs are important in aquaculture, and provide a small yet significant protein contribution to the human diet [66]
echinoderms	juvenile life stages, egg fertilization and early development can be highly vulnerable, resulting in much reduced survival [67–69]. Adult echinoderms may increase growth and calcification; such responses are, however, highly species-specific [45]
crustaceans	the relative insensitivity of crustaceans to ocean acidification [47,70,71] has been ascribed to well-developed ion transport regulation and high protein content of their exoskeletons [43]. Nevertheless, spider crabs show a narrowing of their range of thermal tolerance by approximately 2^°^C under high CO_2_ conditions [72]
foraminifera	shell weight sensitive to 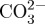 decrease in the laboratory [73] with field evidence for recent shell thinning [21,74]
fish	adult marine fish are generally tolerant of high CO_2_ conditions [51,75,76]. Responses by juveniles and larvae include diminished olfactory and auditory ability, affecting predator detection and homing ability in coral reef fish [77–79], reduced aerobic scope [80] and enhanced otolith growth in sea bass [81]
coralline algae	meta-analysis [43] showed significant reductions in photosynthesis and growth due to ocean acidification treatments. Elevated temperatures (+3^°^C) may greatly increase negative impacts [82]. Field data at natural CO_2_ vents show sensitivity of epibiont coralline algae [83,84]
non-calcified macro-algae; sea grasses	both groups show capability for increased growth [42,43]. At a natural CO_2_ enrichment site, sea grass production was highest at mean pH of 7.6 [83]
coccolithophores	most studies have shown reduced calcification in higher CO_2_ seawater, as first found by [85]. However, the opposite effect has also been reported [86], and ocean acidification impacts on coccolithophore photosynthesis and growth are equivocal, even within the same species. This variability may be due to the use of different strains [87], experimental conditions [88] and species-specific sensitivities to different carbonate chemistry parameters [49]
bacteria	most cyanobacteria (including *Trichodesmium*, a nitrogen-fixer) show enhanced photosynthesis and growth under increased CO_2_ and decreased pH conditions [89,90]. Heterotrophic bacteria investigated to date show many responses with potential biogeochemical significance, including decreased nitrification and increased production of transparent exopolymer particles (affecting aggregation of other biogenic material and its sinking rate) [44]. Adaptation to a high CO_2_ world is likely to be more rapid by bacteria and other short-generation microbes than by multicellular organisms [10]

The scaling of this wide range of experimental responses, whether negative or positive, to ecological and biogeochemical impacts is not straightforward [88,89], and many knowledge gaps remain, at the species, community and ecosystem levels. Such uncertainties and ambiguities are in part due to methodological differences that complicate or invalidate intercomparisons (e.g. whether pH is directly measured or computed from other parameters; duration and level of CO_2_ exposure; whether acidification is achieved by CO_2_ enrichment or by adding acid; and the relative availability of nutrients/food) and in part due to the difficulty in carrying out experiments involving multi-species interactions over long time periods, taking account of confounding variables (e.g. temperature, nutrient availability) and the potential for adaptive responses. There is also inherent biological variability, that can be strain-specific [87,88]. This should not be surprising, since the ocean harbours an enormous biodiversity, with strong competitive pressures to exploit the whole range of environmental conditions.

**Figure 3.**
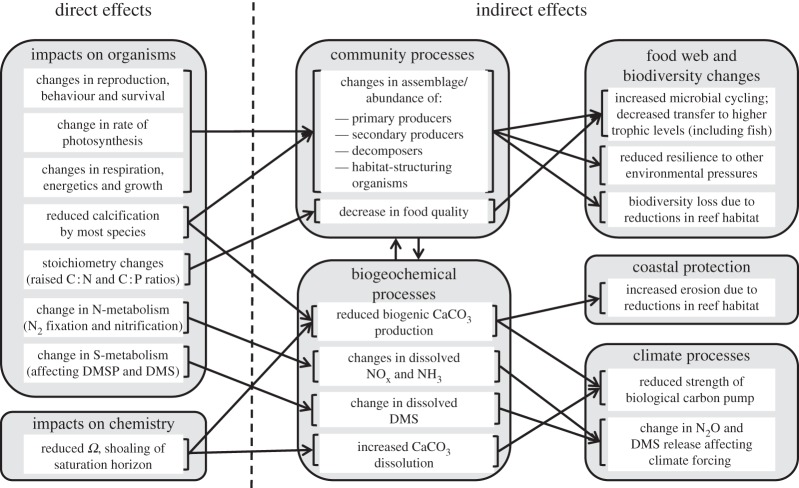
Conceptual representation of possible future ocean acidification impacts on planktonic and benthic organisms, with implications for ecosystems and ecosystem services. DMS, dimethylsulphide; DMSP, dimethylsulphoniopropionate; *Ω*, saturation state (for CaCO_3_). Image: T. Tyrrell and P. Williamson.

Major national and international programmes are currently underway to address these issues. These programmes use standardized protocols [91] to improve intercomparability; they are also attempting to integrate experimental studies, fieldwork and modelling, with effort directed at elucidating genetic and physiological factors that affect both short- and long-term responses. The overall goal is to assess ocean acidification impacts from the molecular to global level, involving studies not only of direct effects on organisms, but also of the potential for indirect effects on biodiversity, climate and socio-economic systems (figure 3).

## 4. Effect of emission reduction on ocean acidification

**Figure 4.**
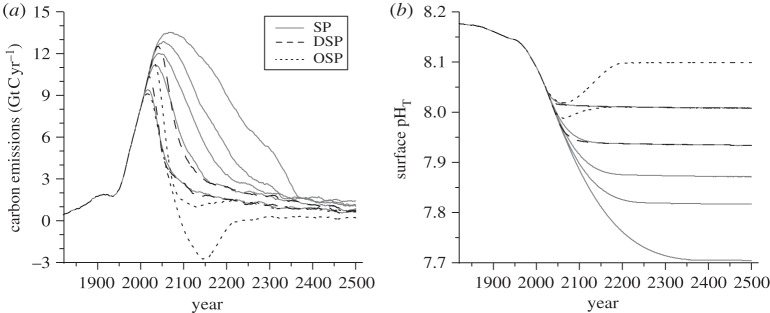
(*a*) The relationship between changes in global annual carbon emissions over the period 1800–2500 and (*b*) global mean surface pH. The pH stabilization levels of 8.10, 8.01, 7.94, 7.87, 7.82 and 7.70 correspond to atmospheric CO_2_ levels of 350, 450, 550, 650, 750 and 1000 ppm. Dotted lines labelled OSP (overshoot stabilization profile) show pathways requiring negative CO_2_ emissions (i.e. carbon dioxide removal geoengineering) to achieve atmospheric CO_2_ stabilization at 350 and 450 ppm; dashed lines labelled DSP (delayed stabilization profile) show delayed approach to emissions reductions to achieve stabilization at 450 and 550 ppm; solid lines labelled SP represent stabilization profiles. From Joos *et al.* [34], modified by permission of Oxford University Press.

The tight relationship between atmospheric CO_2_ and surface ocean chemistry means that emission reduction measures that stabilize the former, e.g. at 450, 550, 650, 750 or 1000 ppm, will also stabilize surface ocean pH, at approximately 8.01, 7.94, 7.87, 7.82 and 7.71, respectively (figure 4) [34]. The predicted consequences of a pH fall of 0.4 (to 7.7, discussed earlier as the ‘business as usual’ scenario) are therefore avoidable, if strong mitigation measures are taken.

In that context, it is valid to ask whether a safe/dangerous threshold can be defined for ocean acidification, in a similar way that a 2^°^C increase (likely to result from an atmospheric level of approximately 450 ppm CO_2_-equivalent) is considered the acceptability threshold, in policy terms, for temperature change [92,93]. Thresholds for dangerous pH change are harder to define, since impacts seem likely to be incremental and regional, rather than involving a single, global-scale ‘tipping point’; furthermore, their economic consequences are currently not well quantified [33,94,95]. Nevertheless, the CO_2_ stabilization target of 450 ppm would still involve considerable risk of large-scale and ecologically significant ocean acidification impacts for the upper ocean.

Thus, at that level: 11 per cent of the global ocean would experience a pH fall of more than 0.2 relative to pre-industrial levels [32]; only 8 per cent of present-day coral reefs would experience conditions considered optimal for calcification (*Ω*_aragonite_>3.5), compared with 98 per cent at pre-industrial atmospheric CO_2_ levels [32]; approximately 10 per cent of the surface Arctic Ocean would be aragonite-undersaturated for part of the year [7]; and potentially severe local impacts could occur in upwelling regions and coastal regions [31,96,97].

The response of deep ocean chemistry to atmospheric CO_2_ stabilization involves very different time scales. Modelling studies indicate that recovery to major perturbations in the global carbon cycle can take 50 000–100 000 years, involving equilibration with carbonate minerals and the carbonate–silicate cycle [98]. Within the next 1000 years, marine CaCO_3_ sediment dissolution is estimated to account for neutralizing 60–70% of anthropogenic CO_2_ emissions, while 20–30% remains in the ocean water column and the remaining approximately 10 per cent is accounted for by terrestrial weathering of silicate carbonates [99].

## 5. Implications of geoengineering for ocean acidification

### (a) General issues

The technological, environmental and socio-economic aspects of geoengineering warrant scientific attention on the basis that, if emission reductions should be insufficient to avert dangerous climate change, other large-scale interventions may need to be seriously contemplated [100–103]. While direct mitigation is the preferred UK and international policy approach, the relatively slow progress to date in global emissions control makes it likely that the ‘safe’ global warming threshold of an approximately 2^°^C increase in global surface temperature (relative to pre-industrial conditions) will be exceeded [104–106].

Differences in the definition of geoengineering have important regulatory implications; e.g. relating to recent decisions by the Convention on Biological Diversity [107]. For considering the implications of geoengineering for ocean acidification, a relatively broad definition has utility, consistent with [100]: ‘the deliberate large-scale manipulation of the planetary environment to counteract anthropogenic climate change’. A key aspect of counteraction is that geoengineering techniques should potentially be capable of remedying future climate problems, i.e. reversing rather than just slowing global warming. Nevertheless, climate stabilization might still be the target outcome (pragmatically achieved in combination with other measures).

The division of geoengineering into solar radiation management (SRM) and carbon dioxide removal (CDR) techniques [100] is now well established, and that terminology is followed here. Other approaches are, however, possible, and these may be given increasing attention in the future; for example: reducing the coverage or long-wave opacity of cirrus clouds [108], and techniques that might actively remove greenhouse gases other than CO_2_ (particularly methane) [109].

Table 2 summarizes the main effects on ocean acidification, as far as they are known, of a range of proposed geoengineering techniques, both SRM and CDR. Additional details are given below.

**Table 2.** Summary of probable main effects of a range of proposed geoengineering approaches on ocean acidification, assuming climatically significant deployment and in comparison to unabated CO_2_ emissions. Within approaches, there may be relatively large differences in effects depending on specific details of techniques and their deployment arrangements. Additional details in text. OA, ocean acidification; SRM, solar radiation management; CDR, carbon dioxide reduction.

	SRM or CDR	approach	probable effects and comments
1. Techniques that either might slightly ameliorate, or slightly worsen, future OA or have no net effect	SRM	space-based reflection	stabilized temperatures but increased CO_2_ expected to have adverse direct impact on OA due to effects on solubility of CO_2_ and CaCO_3_. However, the comparison is complicated by temperature effects on terrestrial carbon biomass in the non-SRM control [110,111]; direct SRM impacts on the hydrological cycle [112]; and the potential for second-order impacts (e.g. irradiance- and CO_2_-induced changes in terrestrial and marine primary production) [113]
	neither	cirrus cloud manipulation	
	SRM	stratospheric aerosols (SO_2_)	as above, plus effect of decreased pH of precipitation (although likely to be slight [114])
	SRM	marine cloud brightening	temperature/solubility effects, with increased likelihood of significant impact due to decreased marine primary production due to change in light quantity and quality [115]
	SRM	ocean surface albedo	
	SRM	land surface albedo	temperature/solubility effects, that may vary inter-hemispherically due to asymmetric SRM cooling [116]
2. Techniques that displace OA from ocean surface to mid- or deepwater	CDR	direct ocean fertilization	additional primary production and carbon export would reduce OA in upper ocean but decrease pH in ocean interior. On century-scale, potential for modest net benefit (due to enhanced CaCO_3_ dissolution at depth) [117–119]
	CDR	up/downwelling modification	
	CDR	direct air capture with ocean storage	potential for severe local OA impacts at site of liquid CO_2_ injection (both midwater and seafloor have been proposed) [120,121]. Long-term fate of injected CO_2_ may be highly location-sensitive [122]
3. Techniques that might counteract OA globally, but with some risk of locally severe deepwater impacts	CDR	direct air capture with sub seafloor storage	small risk of potentially severe OA impacts due to reservoir failure [123–125]; such risks might be reduced if CO_2_ injected into basaltic rocks [126]
	CDR	ocean storage of terrestrial biomass	very slow decomposition (with low CO_2_ release and OA impacts) could be achieved if biomass (e.g. crop waste) is deposited in high-sedimentation sites; e.g. off major river-mouths [127]
4. Techniques that, in theory, could counteract OA, if achievable at necessary scale	CDR	enhanced ocean alkalinity	could directly ameliorate OA at ocean surface, but with local risk of high pH/alkalinity impacts [128–131]. Range of techniques proposed, with most likely to be slow acting [132]
	CDR	enhanced soil alkalinity	river run-off of minerals and enhanced alkalinity could have second order OA impacts (negative or positive) for coastal areas
	CDR	afforestation/ reforestation	if successful in reducing atmospheric CO_2_, would also reduce future OA without significant unintended side effects on ocean chemistry
	CDR	biochar and other techniques to enhance soil C	
	CDR	direct air capture with land-based geological storage	

### (b) Solar radiation management

SRM techniques (also known as sunlight reflection methods) are intended to decrease the amount of solar irradiance reaching the Earth, by increasing the albedo (reflectivity) of the upper, mid or lower atmosphere, or of the land or ocean surface. The main implications of SRM for ocean acidification are relatively straightforward, since atmospheric CO_2_ (and hence ocean chemistry changes) would continue to be primarily determined by CO_2_ emissions.

However, there may also be significant secondary effects of SRM on the ocean carbonate system, driven by larger-scale environmental changes involving temperature, light and other factors. Several modelling studies have assessed the climatic consequences of both atmosphere-based and surface-based SRM (e.g. [133–135] and [116,136], respectively), and an international model inter-comparison exercise is currently underway [112,137]. Such studies have clearly demonstrated that SRM techniques are potentially able to counteract anthropogenic radiative forcing at the global scale due to greenhouse gases. Yet the detailed implications of SRM geoengineering for ecosystems and global carbon dynamics are complex and uncertain [138,139], being a function of SRM techniques and their spatial application (affecting regional patterns of temperature and precipitation, and the frequency of extreme events), and also what scenarios are used as ‘control’ comparisons to quantify the SRM impact. Furthermore, temporal aspects of SRM implementation can also be important: different outcomes in terms of biogeochemical changes on land and in the ocean are likely to result from SRM when applied (i) under present-day conditions (to achieve a global surface cooling of X^°^C in, say, the next 5 years); or (ii) over a multi-decadal time scale to stabilize temperatures (i.e. preventing an increase of X^°^C over 50 years); or (iii) in 50 years time, that might be attempted to restore temperatures to present-day conditions (reversing an increase of X^°^C after it had occurred).

Assessment of ocean acidification responses to SRM-driven temperature change not only requires relatively straightforward information on CO_2_ and CaCO_3_ solubility in the upper ocean (with cooler temperatures having the net effect of decreasing pH), but also understanding of the much more complex climatic impacts on natural carbon sinks and sources [110]. Only one modelling experiment has to date explicitly explored the implications of such interactions for ocean chemistry [111]: that experiment showed that globally uniform atmospheric SRM (to maintain pre-industrial surface temperatures) might reduce the increase in atmospheric CO_2_ by approximately 110 ppm in comparison to an A2 emissions scenario, due to avoidance of climatic impacts on terrestrial biomass (i.e. preventing the net release of biogenic CO_2_ in addition to anthropogenic emissions). That biospheric CO_2_ response contributed to a net increase in global ocean surface pH by 0.05 units, compared with the A2 control, although with no effective change in aragonite saturation state [111].

Two other second-order consequences of atmospheric-based SRM geoengineering for ocean acidification are also possible, but have yet to be explored in modelling experiments.

— SRM-induced changes in light quality and quantity could affect primary production, and hence other aspects of carbon dynamics in the atmosphere and the ocean. While terrestrial vegetation might be more productive under diffuse light conditions [113], that effect is inherently less likely for marine phytoplankton—although it has yet to be quantitatively assessed.
— CO_2_-induced ocean acidification could be exacerbated if sulphate aerosols were used for SRM, due to their effect on precipitation pH. Such impacts would probably be slight, since the quantity of sulphur that, in theory, would need to be added to the stratosphere for geoengineering (1–5 million tonnes per year) [140] is at least an order of magnitude less than that currently added to the total atmosphere by industrial activities and volcanic emissions [100].

As initially stated, the overall consequence of SRM is that ocean acidification will continue, despite the complexity of interactions identified above. Marine organisms would therefore continue to experience ocean acidification impacts under SRM; they would, however, benefit by only being subject to a single stress, since deleterious temperature increase would have been averted (assuming SRM effectiveness).

Most experimental studies on ocean acidification carried out to date have not changed temperature as an additional experimental treatment. For those that have, impacts have generally been greater when both stresses are applied [63,72,80], yet with exceptions [141,142]. Interpretation of such studies is not straightforward, since (i) sensitivity to temperature change can vary greatly with season and life-cycle stage; (ii) synergistic effects between ocean acidification and temperature may occur [143], although well-controlled experiments are needed to conclusively demonstrate such interactions [144]; (iii) species may have different adaptive capabilities (physiological and genetic) in response to ocean acidification and temperature changes, particularly on decadal to century time scales; and (iv) marine species could be expected to change their geographical distributions in response to future global warming, but less easily (if at all) in response to ocean acidification.

### (c) Carbon dioxide removal

Geoengineering based on CDR aims to constrain global warming by directly counteracting CO_2_ emissions, thereby increasing the likelihood of stabilization of atmospheric CO_2_, preferably at a non-dangerous level. The international policy target [92,93] of 450 ppm CO_2_ will be extremely difficult to achieve by emission reductions alone [105], while the lower target of 350 ppm (proposed on the basis of ecological considerations and to minimize the risk of reinforcing feedbacks [57,145]) has already been exceeded by approximately 40 ppm. Figure 4 shows that net negative emissions are likely to be needed for more than a century (2100–2200), peaking at −3 Gt C yr^−1^ in the middle of that period, in order to achieve surface ocean pH stabilization at 8.1, corresponding to atmospheric CO_2_ stabilization at 350 ppm.

CDR-based geoengineering might seem well suited to directly address both climate change and ocean acidification. Yet two provisos are necessary. First, few CDR techniques would seem sufficiently scalable to be able to counteract more than approximately 50 per cent of current greenhouse gas emissions, and many might only manage less than or equal to 10 per cent [132,146,147]. Thus only modest amelioration of global warming and ocean acidification might be achievable. Further consideration of such techniques as potential geoengineering options could therefore only be justified in the context of a ‘multi-wedge’ policy also involving strong mitigation [148], or if they also deliver other benefits. Second, some ocean-based CDR techniques (if capable of being implemented on a large enough scale) might relocate the process of ocean acidification from the sea surface to midwater or at depth. Such aspects are summarized in table 2, and discussed on a technique-specific basis below.

Chemically based CDR technique (i.e. direct air capture) is considered theoretically capable of removing CO_2_ from the atmosphere at the multi-gigaton scale [149]. It does, however, require that safe, long-term storage of CO_2_ is achievable, for which sub-seafloor sequestration of liquid CO_2_ is generally favoured [123]. This technique is already in use at pilot scale, as a component of at-source CO_2_ removal (climate change mitigation through carbon capture and storage) [124] with marine geological disposal subject to international regulation through the London Convention/London Protocol. In the event of reservoir failure, risk to benthic ecosystems from local acidification could be severe [121,125,150]. Nevertheless, the likelihood of leakage is considered low, provided that the CO_2_ is stored in deep geological strata with impermeable cap rocks, and impacts arising from leakage would be local [150], arguably comparable in scale to existing natural seafloor CO_2_ emissions [83,151].

A range of other potential CDR techniques involve more direct dependence on ocean storage, ocean-based enhanced weathering, or other ocean processes. Proposed storage options include adding liquid CO_2_ to midwater at a depth of approximately 1500 m [120]; forming CO_2_ lakes on the seafloor [152]; or adding carbon to the deep-sea floor in organic form, as baled crop residues [123,153]. All these techniques are, in theory, capable of reducing the rate of increase in atmospheric CO_2_ and thereby rate of ocean acidification in the upper ocean; however, they transfer the problem to mid- or deepwater, with a high risk of acute local impacts and more diffuse, long-term changes in carbonate chemistry on a regional and, ultimately, global basis. The effectiveness of midwater CO_2_ disposal for carbon sequestration is likely to vary considerably between different ocean basins, and is also sensitive to injection depth [122].

The use of ocean-based enhanced weathering [128] could more directly counter ocean acidification, increasing atmospheric CO_2_ drawdown through the addition to the ocean of either bicarbonate [129], carbonate minerals [130], calcium hydroxide [131] or combining the addition of liquid CO_2_ to the ocean with pulverized limestone [154]. All these approaches, however, involve the transport and processing of considerable bulk of materials, with associated energy costs, in order to achieve globally significant climate benefits. The land-based production of Ca(OH)_2_ would also require additional CO_2_ sequestration effort (to avoid additional CO_2_ release), while the various processes proposed for ‘liming the ocean’ could themselves cause large-scale ecosystem damage, by locally raising pH beyond organisms’ tolerance limits and/or decreasing light penetration, through precipitation effects.

Ocean fertilization is a relatively well-studied and assessed [155] CDR option, based on increasing biological productivity by directly adding nutrients, particularly iron [156,157], or increasing their internal re-supply, through enhanced upwelling or downwelling [158,159]. However, only a small proportion of the biologically fixed carbon is removed from circulation on a long-term basis, limiting the effectiveness of ocean fertilization as a CDR option, and there are risks of unintended impacts, e.g. N_2_O release [155]. Most of the increased export of organic carbon from the surface ocean would subsequently be decomposed in mid- and deepwater; thus pH decreases, carbonate chemistry changes and ecosystem impacts are re-located to those depths. Subsequent mixing in the ocean interior and return of deep waters to the surface via upwelling would mean that surface waters would eventually also experience ocean acidification due to the CDR intervention.

On a century-long time scale, it is estimated [160] that iron-based, global-scale ocean fertilization could achieve a maximum atmospheric reduction of approximately 33 ppm CO_2_, while counteracting surface ocean acidification by 0.06 pH units [117]. The Southern Ocean is the area where iron-based geoengineering would be most effective [160]; however, the likelihood of such action being implemented there is reduced by three factors.

— There is poor understanding of natural iron-supply mechanisms in that area, and how they might alter in future (with potentially large changes to cryospheric processes). Those uncertainties would affect verification and impact monitoring for large-scale fertilization [161]; e.g. use of satellite imagery to distinguish iron-induced blooms from natural ones.
— The sea conditions (and logistics) of the Southern Ocean are inimical for large-scale operational deployments.
— There is special conservation protection for the Southern Ocean south of 60^°^ S, via the Protocol on Environmental Protection to the Antarctic Treaty (also known as the Madrid Protocol), that would require international amendment to allow geoengineering to proceed.

The enhancement of land-based carbon sequestration, e.g. by biochar or other techniques to increase soil carbon, is not expected to have significant unintended consequences for ocean acidification (table 2)—and might be politically more acceptable. However, the overall effectiveness of such land-based CDR techniques remains uncertain [100,146,147].

## 6. Conclusions

The chemical process of ocean acidification (pH reduction) is a certain consequence of increasing atmospheric CO_2_ and is already occurring on a global scale, particularly in near-surface waters. While the biological and ecological consequences of the ocean acidification that has occurred to date are considered relatively slight, serious consequences for ecosystems (and ecosystem services) seem inevitable on decadal-to-millennial time scales if CO_2_ emissions continue on current trajectories.

Climate geoengineering through SRM will not affect levels of anthropogenic CO_2_ in the atmosphere, and ocean acidification will therefore continue. However, large-scale deployment of SRM would not restore the global climate to its pre-industrial state, and is likely to result in second-order effects on Earth system processes—with implications for the global carbon cycle, and hence atmospheric CO_2_ and ocean acidification. The magnitude, and even direction, of such effects is currently uncertain: not only are they highly technique-specific, but they will anyway differ according to which projected emissions pathway (and global climate model) is used for the comparison.

CDR techniques are more closely directed at counteracting anthropogenic climate change due to greenhouse gas emissions; they may also provide a more politically acceptable means of tackling the threat of dangerous climate change. Their implications for ocean acidification are also technique specific: while some (in theory) permanently remove carbon from circulation, others re-locate and redistribute the problem of excess CO_2_ from the atmosphere and upper ocean to mid- or deep water. Moreover, CDR techniques proposed to date seem relatively ineffective in terms of the maximum reduction in atmospheric CO_2_ that they might realistically achieve.

The potential for some CDR techniques would seem to warrant further consideration. Nevertheless, strong and rapid mitigation measures, to stabilize atmospheric CO_2_ at near-current levels, would provide the policy action most likely to limit ocean acidification and its associated impacts.
